# Depletion a Cure for Gangrena Senilis

**Published:** 1829

**Authors:** 


35. *Depletion a cure for Gangrena Senilis.*—As far as we know the prevailing treatment of this malady, in America, is that of Mr. Pott—the liberal administration of opium. But one case has presented itself in our practice, and it proved fatal under the aforesaid method. In the Medico-Chirurgical Review for Jan. 1829, we find some account of a memoir on this disease, by Dr. Victor Andry of Paris, which we think deserves to be transferred to our columns. By the way, we may remark, that gangrene, in all its varieties, is far less common in Cincinnati, than it was twenty or twenty-five years ago. To what cause or causes, this should be ascribed we are not prepared to say. It may, perhaps, be owing to improved modes of treating inflammation, or to a change in the occult qualities or constitution of the air, or to both. Of one cause only can we speak with the confidence of being correct. It is the more sparing application of *blisters.* We can recollect when these cruel applications were resorted to with the greatest recklessness, in states of high vascular excitement and exalted sensibility; in the early stages of acute inflammation; in hopeless organic affections; and in conditions of exhaustion and extreme debility. Under all these circumstances, the blistered surface was liable to mortification. But we must return to the promised extract.

Doctor Victor Andry has lately published a long memoir on Gangrene, and more especially the spontaneous, or Senile Gangrene, described by Pott, and other writers.

The Doctor undertakes to demonstrate that all gangrenes, under whatever form they present themselves, are referrible to one proximate cause—the cessation of the circulation in the part affected. All the occasional causes of gangrene, he maintains, act in the same manner, namely, by producing a mechanical obstruction to the course of the blood, or inflammation of the vessels themselves. This last, he avers, is the most frequent cause of gangrene, and that by interruption of the circulation. The experiments of Kaltenbrunner, and many others, prove that, in all inflammations, there is a portion of the part inflamed, in which the circulation is nearly, if not quite stagnant. If the inflammation be very violent, or if, from some other cause, the circulation be not re-established in the minute vessels, gangrene, of more or less extent ensues.

In respect to the gangrena senilis, or Pott's gangrene, Dr. Andry observes that the cessation of the circulation, in consequence of inflammation of the vessels, has not been generally acknowledged as the cause. Although the disease is certainly much more frequent among old than among young people; yet it is occasionally seen in all periods of life—and, in all cases, he maintains, it is owing to inflammation of the veins. M. Baffos lately communicated to the Royal Academy of Medicine, the case of a young woman, twenty years of age, affected with dry gangrene of the feet, attended with excruciating pains, but without any change in the color or in the temperature of the skin. On dissection, the veins of both the lower extremities were found inflamed, and filled with a kind of substance that adhered most tenaciously to the internal surface of the vessels. M. Leveille has reported a somewhat similar case. The spontaneous gangrene was seated in the left leg. On dissection, they found the external iliac artery and the crural vein intensely inflamed and very much thickened, being lined with a fibrinous substance which did not, however, obliterate the calibre of the vessels.

The doctrine of ossification of the arteries, as the cause of gangrena senilis, is not always borne out by facts. Bichat has shewn that, in few individuals, after the age of 60 years, are there wanting traces of ossification of the arteries. In fact, there are many cases recorded, where the arteries of limbs were completely ossified, without any symptom of gangrene being present.

Without stopping to examine the spmptoms and progress of this painful and generally fatal disease, we shall come at once to the principles of treatment—principles which flow naturally from the proximate cause here laid down.

"When then," says the author, "severe pains have preceded the appearances of gangrene, with hard and full pulse, watchfulness,&c. we may conclude that there is inflammation of the arterial or venous coats, and we ought to have recourse to blood-letting, both local and general, if the strength of the patient will permit, together with stimulating frictions and fomentations on the surface of the parts threatened with gangrene."

The following case, communicated by M. Dupuytren, from the Hotel Dieu, will put the good effects of depletion in a clear point of view.

*Case.* A female, aged upwards of sixty years, was received into the Hotel Dieu, and there remained nearly a year, for the treament of Gangrena Senilis, affecting the toes of the left foot. Acute and long continüed pains had preceded the gangrene, and deprived the patient, for some months, of sleep. The toes of the foot were like those of a mummy, while the neighboring parts were of a violet color, and emitted an insupportable smell. For the first few months of the patient's sojourn in the Hospital, opium, bark, and a variety of remedies were tried, without the least benefit. On the contrary, the disease made progress. The whole foot, soft and hard parts, were stricken with gangrene. The state of the heart, the lungs, and the great arteries was carefully examined, but no deviation from healthy function could be discovered, M. Dupuytren now, being tired with the various anodyne and tonic remedies usually employed, determined on opposite measures. Eight ounces of blood were taken from the arm. The pains were mitigated—and some sleep was procured. In fact, the patient experienced more relief from this bleeding, than from all the other remedies put together. This amelioration lasted nearly a fortnight, when the pains began to resume their former severity. The bleeding was repeated, and with the same good effects as before. After this, phlebotomy was had recourse to, whenever the symptoms were distressing; and by this measure, the progress of the gangrene was arrested, the mortified parts separated, and the patient was discharged cured.

Since the above period, a great many people affected with gangrena senilis have been treated in the same manner, and always with success. M. Dupuytren concludes by recommending the depletory treatment, whenever the disease is attended with severe pain, considerable tumefaction of the neighboring parts, fulness and hardness of the pulse, and flush of the face. We think there can be no doubt that this is sound advice.—*Journal de Progres.*

